# Supplementary material for: mRNA therapy restores euglycemia and prevents liver tumors in murine model of glycogen storage disease
Source: Nat Commun. 2021 May 25;12:3090. doi: 10.1038/s41467-021-23318-2 (PMC8149455; doi:10.1038/s41467-021-23318-2)
Supplement: Supplementary file 1 — Supplementary Information [file 41467_2021_23318_MOESM1_ESM.pdf]

SUPPLEMENTARY MATERIALS

Title: mRNA therapy restores euglycemia and prevents liver tumors in murine model of glycogen storage disease

Cao et al.

SUPPLEMENTARY METHODS

Glycogen quantification

Mouse liver sections were stained with standard Hematoxylin and Eosin (H&E) protocol. Images were captured with the Panoramic 250 Flash II (3DHISTECH, Budapest, Hungary) digital slide scanner. For quantification of glycogen level, full slide image analysis was performed using an algorithm with classifiers through HALO software.

Detection of h*G6PC* S298C mRNA by *in situ* hybridization

Mouse livers were trimmed and fixed in 10% neutral buffered formalin (NBF) and underwent standard paraffin processing, embedding, and sectioning (5µm thickness). *In situ* hybridization was performed using the Leica Bond RX autostainer (Leica Microsystems, Buffalo Grove, IL). Liver sections were baked and deparaffinized on the instrument, followed by the RNAscope 2.5 LSx DAB ISH protocol using the RNAscope 2.5 LS reagent kit-brown (cat#322100) in which the target probe signal is visualized by chromogenic diaminobenzidine. ISH Probes used were RNAscope® LS Positive Control Probe mmPPIB (cat#313928), RNAscope® 2.5 LS Negative Control Probe dapB (cat#312038), and a proprietary probe to our h*G6PC* S298C construct.

Hepatocellular adenoma (HCA) prevention study

RT-qPCR

For RT-qPCR analysis, frozen liver fragments were processed by RNAlater™-ICE Frozen Tissue Translation Solution, followed by total RNA isolation using Qiagen miRNeasy Mini Kit (Qiagen, cat #217004). The quality of total RNA was assessed by Invitrogen Quant-iT RNA Broad Range Assay Kit (Invitrogen, cat #Q10213). RT-qPCR experiment was performed on the isolated RNAs (10ng/ul) in triplicates, using TaqMan™ Fast Virus 1-Step kit (ThermoFisher, cat# 4444432) on an ABI QuantStudio 7, following manufacturer’s recommended cycling conditions. The Taqman probes used for quantification of mouse mRNA transcripts are as

follows: *Ctnnb1*, Mm00483039_m1; *Tgfb1*, Mm01178820_m1; and *Glul*, Mm00725701_s1. All results are represented as ΔΔCt and normalized by housekeeping gene Glyceraldehyde 3-phosphate dehydrogenase (Gapdh) quantified by the probe Mm99999915_g1.

ELISA

Serum levels of mouse AFP were measured with commercially available ELISA kits from Abcam (AFP cat #210969) by following manufacturer's instructions.

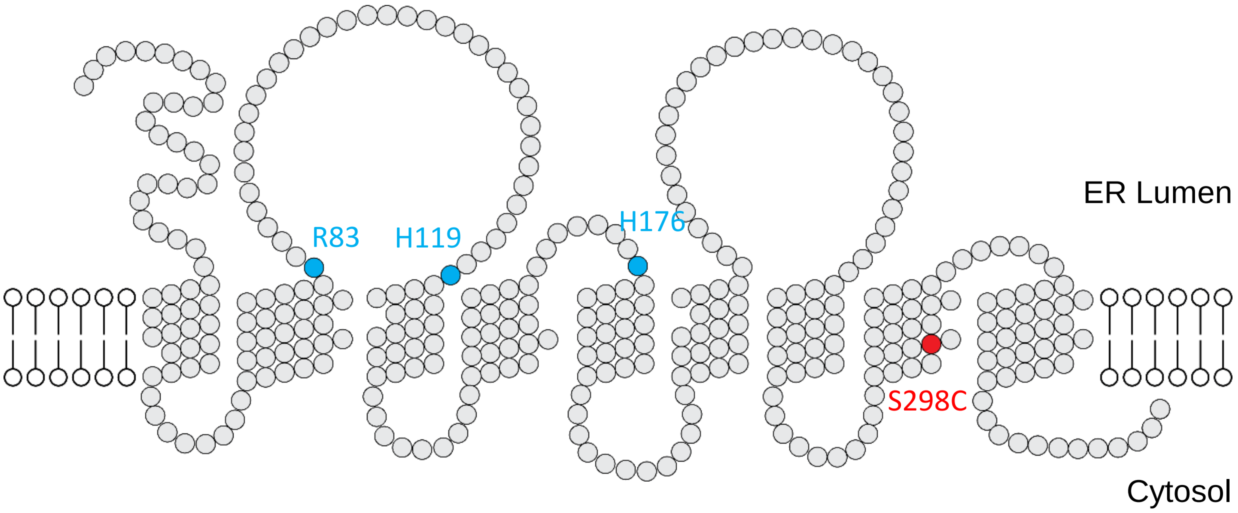

Supplementary Fig. 1. Predicted topology of hG6Pase-α. hG6Pase-α is a nine-transmembrane spanning enzyme that resides in the ER membrane. Blue circles: residues directly involved in enzymatic activity. Red circle: location of S298C modification.

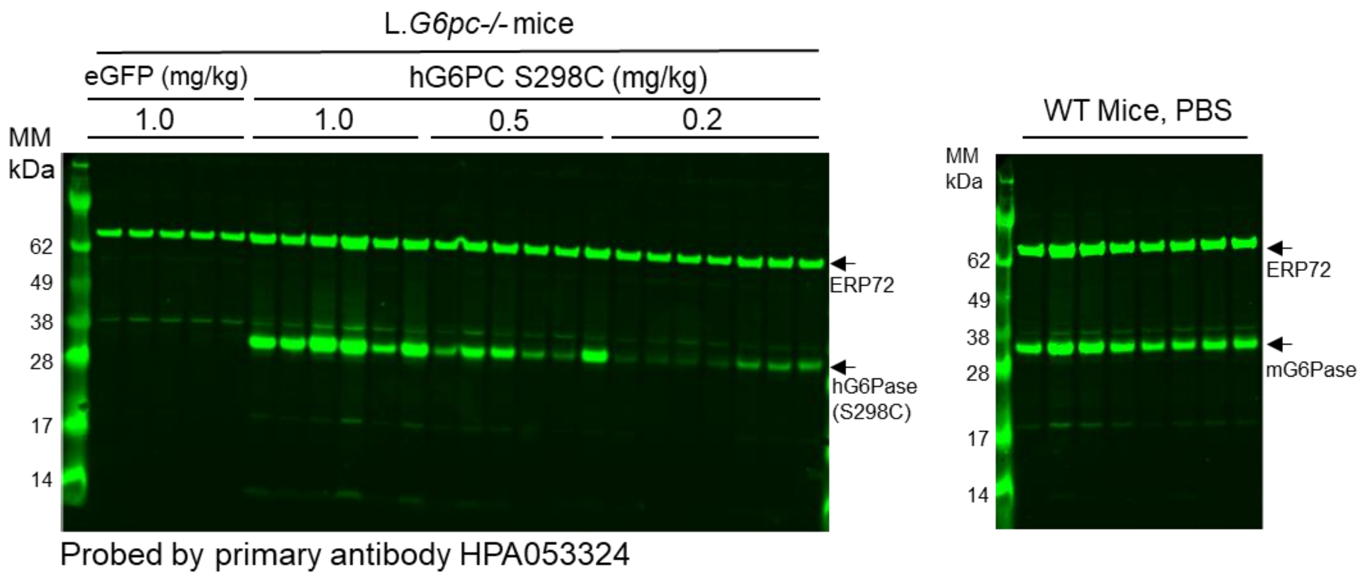

Supplementary Fig. 2. hG6Pase-α S298C protein expression in livers of L.*G6pc*-/- mice measured by immunoblot analysis. Immunoblots of data from dose ranging study in Fig. 4c. Liver microsomes derived from L.*G6pc*-/- male mice treated with either eGFP or h*G6PC* S298C mRNA/LNP (left) or from WT male mice treated with PBS (right). The blots were probed with anti-G6Pase antibody HPA053324 with ERP72 as a loading control. The experiment was repeated independently for two times, with similar results.

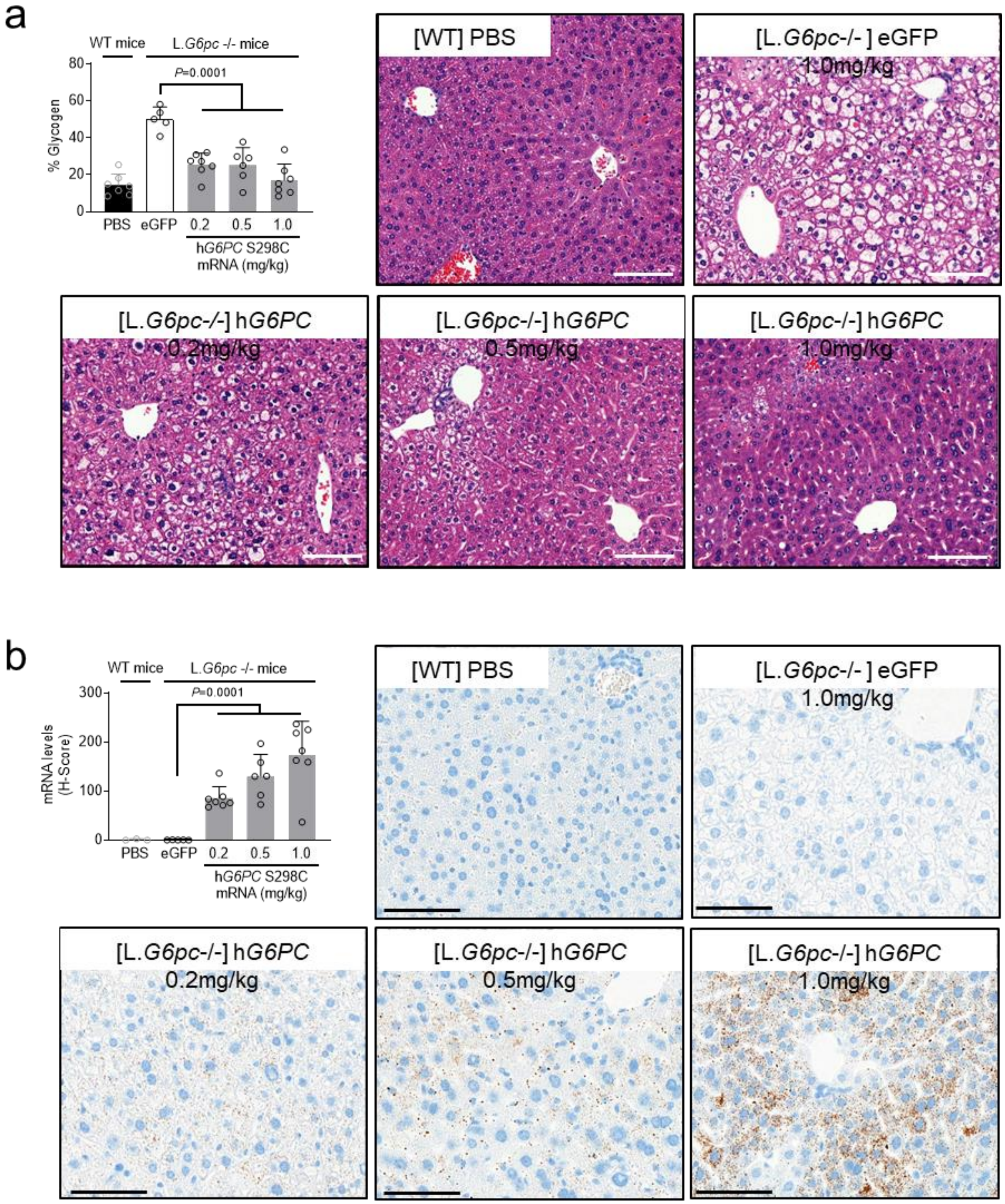

Supplementary Fig. 3. Histological analysis of *L.G6pc-/-* mouse livers treated with h*G6PC*-S298C mRNA-LNP injected i.v. a) Hepatic glycogen measured by H&E staining. **b)**

h*G6PC*-S298C mRNA detected by *in situ* hybridization. For both **a** and **b**, Representative images and quantitative analysis in bar graph are shown as mean ±SD (n = 7, 5, 7, 6, and 7 mice per group from WT treated with PBS, L.*G6pc*-/- treated with eGFP, or L.*G6pc*-/- treated with h*G6PC* S298C mRNA at 0.2, 0.5, or 1.0 mg/kg, respectively). For statistical analysis, raw values were subjected to one-way ANOVA, followed by the Dunnett's multiple comparisons test, compared to the eGFP mRNA treated group. *P* values are shown in the graphs. Scale bars are 100 µm. Source data are provided as a Source Data File.

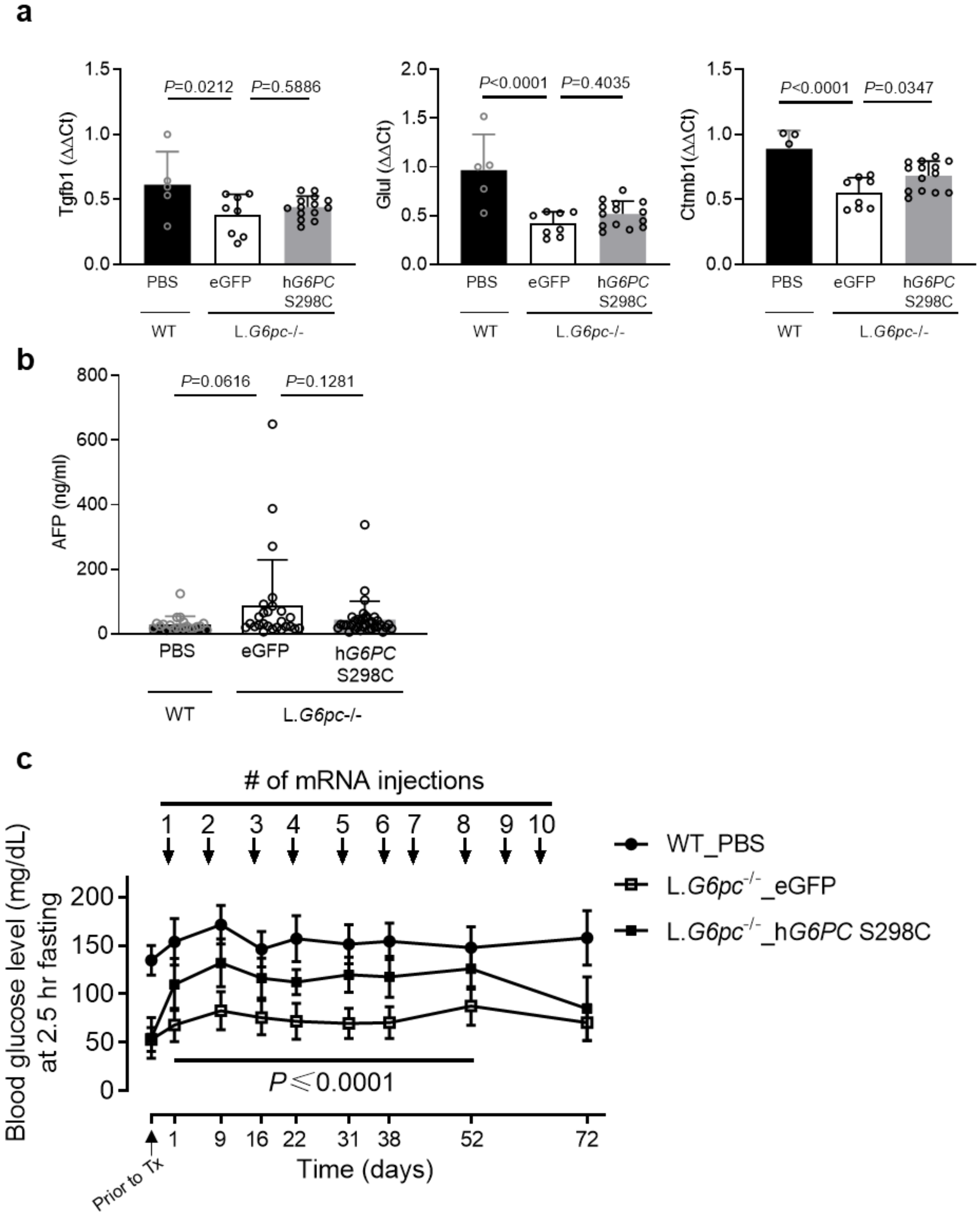

Supplementary Fig. 4. Effect of h*G6PC* S298C mRNA-LNP on prevention of hepatic adenomas in L.*G6pc-/-* mice. a) mRNA levels of hepatic biomarkers involved in cell proliferation and HCA/HCC (*Tgfb1, Glul, and Ctnnb1*). Results are expressed as mean ±SD (n = 5, 8, and 14 mice per group for WT treated with PBS, L.*G6pc-/-* treated with eGFP, or L.*G6pc-/-*

treated with h*G6PC* S298C mRNA). **b)** HCA/HCC-related serum biomarker alpha fetal protein (AFP). Results are expressed as mean ±SD (n = 21, 26, and 34 mice per group for WT treated with PBS, L.*G6pc*-/- treated with eGFP, or L.*G6pc*-/- treated with hG6PC S298C mRNA). **c)** Fasting blood glucose levels (2.5 hrs post-fasting). Results are expressed as mean ±SD (n = 17, 23, and 23 mice per group for WT treated with PBS, L.*G6pc*-/- treated with eGFP, and L.*G6pc*-/- treated with h*G6PC* S298C mRNA, respectively). For statistical analysis, raw values were subjected to one-way ANOVA, followed by the Dunnett's multiple comparisons test, compared to the eGFP mRNA treated group. *P* values are shown in the graphs. Source data are provided as a Source Data File.

GGGAAAUAAGAGAGAAAAGAAGAGUAAGAAGAAAUAUAAGAGCCACCAUGGAGGAGGGCAUGAACGUGC
UUCACGACUUCGGCAUCCAGUCAACCCAUUACCUACAGGUGAACUAUCAGGACUCCCAGGAUUGGUUCA
UCCUCGUGAGCGUGAUCGCCGAUCUAAGAAACGCAUUCUACGUGUUGUUCCCAAUCUGGUUCCACCUGC
AGGAAGCUGUGGGCAUCAAGCUCCUUUGGGUGGCCGUGAUUGGCGAUUGGCUGAACCUGGUGUUCAAGU
GGAUUCUCUUCGGUCAGCGGCCUUACUGGUGGGUGUUGGAUACCGACUAUUACAGUAACACCUCUGUUC
CGCUGAUCAAGCAGUUCCCUGUCACCUGCGAAACCGGACCUGGCUCCCCUUCCGGCCACGCCAUGGGCA
CCGCCGGCGUGUACUACGUGAUGGUGACAUCCACCCUGAGCAUCUUCCAGGGCAAGAUCAAGCCUACCU
ACAGAUUCAGGUGCCUGAACGUUAUCUUGUGGCUGGGAUUCUGGGCAGUGCAGCUCAACGUGUGCCUGA
GCAGGAUCUAUCUCGCAGCCCACUUCCCACAUCAGGUCGUGGCCGGCGUACUGAGCGGAAUCGCCGUGG
CUGAAACCUUCAGCCAUAUCCACAGCAUCUACAAUGCCUCCCUGAAGAAGUACUUCCUUAUUACAUUCU
UCCUGUUCUCUUUCGCCAUCGGCUUCUAUCUGCUGCUAAAGGGCCUGGGCGUCGACCUCCUGUGGACCC
UGGAGAAGGCCCAGAGAUGGUGCGAACAACCUGAGUGGGUGCACAUCGACACCACUCCGUUCGCUAGCC
UGCUCAAGAACCUGGGCACUCUUUUCGGCCUGGGCCUGGCCCUCAAUAGCAGCAUGUACCGGGAGAGCU
GUAAGGGAAAGCUGUCCAAGUGGCUCCCUUUCAGACUUAGCUGCAUCGUGGCGUCUCUCGUGCUGCUGC
ACGUGUUCGACAGCCUGAAGCCUCCUUCGCAGGUGGAGCUGGUGUUCUACGUGCUGAGCUUCUGCAAGA
GCGCGGUGGUGCCACUGGCAAGUGUCAGCGUGAUCCCUUAUUGCCUGGCCCAGGUGCUGGGCCAGCCUC
AUAAGAAGUCCCUGUGAUAAUAGGCUGGAGCCUCGGUGGCCUAGCUUCUUGCCCCUUGGGCCUCCCCCC
AGCCCCUCCUCCCCUUCCUGCACCCGUACCCCCGUGGUCUUUGAAUAAAGUCUGAGUGGGCGGCAAAAA
AAAAAAAAAAAAAAAAAAAAAAAAAAAAAAAAAAAAAAAAAAAAAAAAAAAAAAAAAAAAAAAAAAAAA
AAAAAAAAAAAAAAAAAAAAAAAAAAUCUAG

Supplementary Table 1: Complete mRNA sequence of h*G6PC*-S298C. The 5′ and 3′ UTRs are underlined.

amino acid	background probability
A	0.073
C	0.025
D	0.05
E	0.061
F	0.042
G	0.072
H	0.023
I	0.053
K	0.064
L	0.089
M	0.023
N	0.043
P	0.052
Q	0.04
R	0.052
S	0.073
T	0.056
V	0.063
W	0.013
Y	0.033

Supplementary Table 2: Background probability of amino acids, for use in calculating relative entropy.

From Rivoire, O., Reynolds, K. A. & Ranganathan, R. Evolution-Based Functional Decomposition of Proteins. Plos Comput Biol 12, e1004817 (2016).

Name/Targeted genes	Sequence	Catalogue #	Supplier
hG6PC S298C	Forward: 5'-GTGGCTCCCTTTCAGACTTAG-3' Reverse: 5'-GAAGCTCAGCACGTAGAACA-3' Probe: 5'-AAGGAGGCTTCAGGCTGTCGAAC-3'	N/A	ThermoFisher
Catenin beta-1 (Ctnnb1)	N/A	Mm00483039_m1	ThermoFisher
Transforming growth factor beta-1 (Tgfb1)	N/A	Mm01178820_m1	ThermoFisher
Glutamine synthetase (Glul)	N/A	Mm00725701_s1	ThermoFisher
Beta-actin (Actb)	N/A	Mm02619580_g1	ThermoFisher

Supplementary Table 3: Primers and reagents for quantitative RT-PCR analysis.

N/A, not available.
