# Supplementary material for: mRNA therapy restores euglycemia and prevents liver tumors in murine model of glycogen storage disease
Source: Nat Commun. 2021 May 25;12:3090. doi: 10.1038/s41467-021-23318-2 (PMC8149455; doi:10.1038/s41467-021-23318-2)
Supplement: Supplementary file 3 — Reporting Summary [file 41467_2021_23318_MOESM3_ESM.pdf]

nature research

Corresponding author(s): Paloma Giangrande, Paolo Martini
Last updated by author(s): April 13, 2021

Reporting Summary

Nature Research wishes to improve the reproducibility of the work that we publish. This form provides structure for consistency and transparency in reporting. For further information on Nature Research policies, see our Editorial Policies and the Editorial Policy Checklist.

Please do not complete any field with "not applicable" or n/a. Refer to the help text for what text to use if an item is not relevant to your study. For final submission: please carefully check your responses for accuracy; you will not be able to make changes later.

Statistics

For all statistical analyses, confirm that the following items are present in the figure legend, table legend, main text, or Methods section.

n/a	Confirmed	
☐	☒	The exact sample size (*n*) for each experimental group/condition, given as a discrete number and unit of measurement
☐	☒	A statement on whether measurements were taken from distinct samples or whether the same sample was measured repeatedly
☐	☒	The statistical test(s) used AND whether they are one- or two-sided<br>*Only common tests should be described solely by name; describe more complex techniques in the Methods section.*
☒	☐	A description of all covariates tested
☐	☒	A description of any assumptions or corrections, such as tests of normality and adjustment for multiple comparisons
☐	☒	A full description of the statistical parameters including central tendency (e.g. means) or other basic estimates (e.g. regression coefficient) AND variation (e.g. standard deviation) or associated estimates of uncertainty (e.g. confidence intervals)
☐	☒	For null hypothesis testing, the test statistic (e.g. F, t, r) with confidence intervals, effect sizes, degrees of freedom and P value noted<br>*Give P values as exact values whenever suitable.*
☒	☐	For Bayesian analysis, information on the choice of priors and Markov chain Monte Carlo settings
☒	☐	For hierarchical and complex designs, identification of the appropriate level for tests and full reporting of outcomes
☒	☐	Estimates of effect sizes (e.g. Cohen's *d*, Pearson's *r*), indicating how they were calculated

Our web collection on statistics for biologists contains articles on many of the points above.

Software and code

Policy information about availability of computer code

Data collection

Sequences alignment was performed using the multiple sequence alignment software tool MAFFTA v7.407 (https://mafft.cbrc.jp/alignment/software/). The consensus amino acids were visualized using Weblogo v.3.7.1 (https://github.com/WebLogo/weblogo). Absorbance data were acquired by the microplate reader BioTek Synergy H1 operated by the Gen5 Microplate Reader and Imager Software (v2). Protein band signals from western blots were visualized and quantified by Odyssey CLx and accompanying software Image Studio V3.1.4. Image in confocal studies was acquired by the Opera Phenix spinning disk confocal microscope operated with Harmony (Perkin Elmer). Data from Taqman RT-PCR were obtained by an ABI QuantStudio 7.

Data analysis

Images in confocal studies were analyzed by Harmony; all other data analysis were performed by Phoenix® WinNonlin® version 8.0 or GraphPad Prism v.7.01.

For manuscripts utilizing custom algorithms or software that are central to the research but not yet described in published literature, software must be made available to editors and reviewers. We strongly encourage code deposition in a community repository (e.g. GitHub). See the Nature Research guidelines for submitting code & software for further information.

Data

Policy information about availability of data

All manuscripts must include a data availability statement. This statement should provide the following information, where applicable:

- Accession codes, unique identifiers, or web links for publicly available datasets
- A list of figures that have associated raw data
- A description of any restrictions on data availability

The authors declare that all relevant data supporting the findings of this study are available within the article and its Supplementary Information files. Source data are provided with this paper.

Field-specific reporting

Please select the one below that is the best fit for your research. If you are not sure, read the appropriate sections before making your selection.

☒ Life sciences ☐ Behavioural & social sciences ☐ Ecological, evolutionary & environmental sciences

For a reference copy of the document with all sections, see nature.com/documents/nr-reporting-summary-flat.pdf

Life sciences study design

All studies must disclose on these points even when the disclosure is negative.

Sample size	The sample size from in vivo studies were determined based on prior experiments and experience, which were used to determine the minimum number of animals needed for evaluating a significance. For Fig, 2b and Fig. 3 focusing on construct validating and PK studies in wild-type mice, 3-4 mice were used per group. For pharmacological studies designed to evaluate the efficacy in disease model (Figures 4-6), a minimum of 6 model mice per treatment group were used. Required sample size was calculated from power calculations needed to show statistical difference in disease-associated primary biomarkers (i,e, fasting blood glucose) between control and treated groups, using a two-sided t-test. All in vitro experiments in cell culture were replicated and performed at least three times independently. The sample size was shown to be sufficient to measure the differences between groups for the assay used.
Data exclusions	The optimal potency of mRNA/LNP as assessed in our studies requires intravenous (i.v.)delivery of drug product. Therefore, mice that did not receive a complete i.v. injection were determined to be technical deviations and excluded from final data analysis. However, no more than two mice in each treatment arm in any of our studies were excluded. The available number of animals/samples being analyzed in all efficacy studies were fully presented in relevant figures as shown in this paper. All exclusion criteria were pre-established.
Replication	All in vitro experiments in cell culture were replicated for at least three times. Key in vivo findings were replicated in a minimum of two independent studies. All findings were reliably reproduced.
Randomization	The L.G6pc-/- mice were randomly assigned to different treatment groups based on their baseline fasting blood glucose levels. The pretreatment baseline 2.5-hr fasting blood levels were first ranked and the mice were distributed to each treatment groups based on the rank of glucose level.
Blinding	Investigators were blinded to treatment groups during data collection. Where possible, all laboratory parameters (specifically primary disease biomarkers and clinical chemistry parameters) were quantified in a blinded fashion.

Reporting for specific materials, systems and methods

We require information from authors about some types of materials, experimental systems and methods used in many studies. Here, indicate whether each material, system or method listed is relevant to your study. If you are not sure if a list item applies to your research, read the appropriate section before selecting a response.

Materials & experimental systems

n/a	Involved in the study	
☐	☒	Antibodies
☐	☒	Eukaryotic cell lines
☒	☐	Palaeontology and archaeology
☐	☒	Animals and other organisms
☒	☐	Human research participants
☒	☐	Clinical data
☒	☐	Dual use research of concern

Methods

n/a	Involved in the study	
☒	☐	ChIP-seq
☒	☐	Flow cytometry
☒	☐	MRI-based neuroimaging

Antibodies

Antibodies used

Western blotting:
Rabbit anti-human G6Pase-alpha (1:500, cat #HPA052324, Atlas Antibodies)
rabbit anti-human G6Pase-α IgG (1:1000, #ab93857, Abcam)
Rabbit anti-ERp72 mAb (D70D12) (1:1000, Cell Signaling, cat #5033)
Rabbit anti-PKM2 (D78A4) XP® mAb (1:1000, Cell Signaling, cat #4953)
Rabbit anti-β-catenin (D10A8) XP® mAb (1:1000, Cell signaling, cat #8480)
Mouse anti-SQSTM1 / p62 mAb (1:1000, cat #ab56416, Abcam)
All secondary antibodies used in the western blotting analysis were (IR)-labeled goat or donkey antibodies against mouse or rabbit IgG as applicable (Goat anti-rabbit, IRDye® 800CW, cat # 926-32211; goat anti-rabbit, IRDye® 680RD, cat # 926-68073; donkey anti-mouse, IRDye® 800CW, cat # 926-32212; donkey anti-mouse, IRDye® 680RD, cat # 926-68072), which were manufactured by Li-COR Biosciences. The dilution of the secondary antibodies was 1:5000.

Confocal Analysis:
Mouse anti-TOM20 mitochondrial marker antibody (1:50, Cat #612278, BD Bioscience)
Rabbit anti-human G6Pase-alpha (1:50, cat # HPA052324, Atlas Antibodies)
Mouse anti-Calnexin mAb (1:50, cat #66332, Abcam)
All secondary antibodies used in the confocal analysis were goat anti-rabbit Alexa 488 or goat anti-mouse Alexa 647, as applicable (cat # A-11070 and #A-21235, ThermoFisher Scientific). The dilution of the secondary antibody was 1:1000.

Validation	Rabbit anti-human G6Pase-alpha (HPA052324, Atlas Antibodies) was validated by the manufacturer and our lab using HeLa cells transfected with mRNAs encoding mouse and human G6Pase-alpha. All other antibodies used in our studies were validated by the manufacturers, with validation statement available in the manufacturer's website.

Eukaryotic cell lines

Policy information about cell lines

Cell line source(s)	All mammalian cell lines (HeLa and Hep3B) were obtained from ATCC
Authentication	Cells were purchased for use in these studies and no authentication was performed after receipt from suppliers
Mycoplasma contamination	Cell line was tested routinely tested for mycoplasma and confirmed to be negative.
Commonly misidentified lines (See ICLAC register)	None

Animals and other organisms

Policy information about studies involving animals; ARRIVE guidelines recommended for reporting animal research

Laboratory animals	All the liver-specific G6pc knockout and WT mice were on a C57BL6 background strain. The disease model mice used in all efficacy studies were male mice because of the need to use tamoxifen for liver deletion of G6pc. Mice were generally 2-month-old at the initiation of all in vivo studies.
Wild animals	None
Field-collected samples	None
Ethics oversight	All the procedures in the liver-specific G6pc knockout mice and littermates were performed in accordance with the principles and guidelines established by the European Convention for the Protection of Laboratory Animals. All conditions and experiments were approved by the University Lyon I animal ethics committee and the French Ministry of National Education, Higher Education and Research (Permit Apafis numbers: 20821-2019052414026539v2 and 25143-2020041814543626 v1). For laboratory animals used in Moderna facilities, all experimental protocols were approved by the Institutional Animal Care and Use Committees at Moderna and complied with all relevant ethical regulations regarding the use of research animals.

Note that full information on the approval of the study protocol must also be provided in the manuscript.

This checklist template is licensed under a Creative Commons Attribution 4.0 International License, which permits use, sharing, adaptation, distribution and reproduction in any medium or format, as long as you give appropriate credit to the original author(s) and the source, provide a link to the Creative Commons license, and indicate if changes were made. The images or other third party material in this article are included in the article's Creative Commons license, unless indicated otherwise in a credit line to the material. If material is not included in the article's Creative Commons license and your intended use is not permitted by statutory regulation or exceeds the permitted use, you will need to obtain permission directly from the copyright holder. To view a copy of this license, visit http://creativecommons.org/licenses/by/4.0/
